# DNA methylation biomarkers of future health outcomes in children

**DOI:** 10.1186/s40348-020-00099-0

**Published:** 2020-07-09

**Authors:** Shivanthan Shanthikumar, Melanie R. Neeland, Jovana Maksimovic, Sarath C. Ranganathan, Richard Saffery

**Affiliations:** 1 Respiratory and Sleep Medicine, Royal Children’s Hospital, Flemington Road, Parkville, Melbourne, Victoria 3052 Australia; 2 Respiratory Diseases, Murdoch Children’s Research Institute, Melbourne, Australia; 3 Department of Paediatrics, The University of Melbourne, Melbourne, Australia; 4 Epigenetics, Murdoch Children’s Research Institute, Melbourne, Australia; 5 Computational Biology, Peter MacCallum Cancer Centre, Melbourne, Australia

**Keywords:** Pediatrics, DNA methylation, Biomarker, Epigenetics, Precision medicine

## Abstract

Biomarkers which predict future health outcomes are key to the goals of precision health. Such biomarkers do not have to be involved in the causal pathway of a disease, and their performance is best assessed using statistical tests of clinical performance and evaluation of net health impact. DNA methylation is the most commonly studied epigenetic process and represents a potential biomarker of future health outcomes. We review 25 studies in non-oncological paediatric conditions where DNA methylation biomarkers of future health outcomes are assessed. Whilst a number of positive findings have been described, the body of evidence is severely limited by issues with outcome measures, tissue-specific samples, accounting for sample cell type heterogeneity, lack of appropriate statistical testing, small effect sizes, limited validation, and no assessment of net health impact. Future studies should concentrate on careful study design to overcome these issues, and integration of DNA methylation data with other ‘omic’, clinical, and environmental data to generate the most clinically useful biomarkers of paediatric disease.

## Introduction

Precision health (also referred to as precision medicine and personalised medicine) can be defined as interventions “targeted to the needs of individual patients on the basis of genetic, biomarker, phenotypic, or psychosocial characteristics that distinguish a given patient from other patients with similar clinical presentations” [1]. It is a rapidly growing field [2] and has been described as an “ongoing revolution in medicine, moving it from a reactive to a proactive discipline, where ultimately the objective is to maximize wellness for each individual rather than simply to treat disease” [3]. Accordingly, significant resources are being allocated to the development of precision approaches across a wide range of healthcare areas with the aim of making discoveries which can be incorporated into routine care [4].

Biomarkers that allow prediction of future health outcomes are key foci of precision health [5]. These may identify those who are at high risk of developing a disease or alternatively may predict disease progression and severity, treatment response, or risk of complications. Advances in genomics in particular, along with bioinformatics, proteomics, and metabolomics, are all contributing to the identification of biomarkers in the precision health field [1]. Whilst biomarkers may be involved in disease pathogenesis, this is not a requirement for them to be clinically useful. Biomarkers have been divided into ‘descriptive’ vs. ‘mechanistic’ biomarkers, where descriptive biomarkers are associated with disease but not directly involved in the causal pathway, and mechanistic biomarkers are directly involved in disease pathogenesis [6]. Either type of biomarker is relevant to the goals of precision health. The utility of biomarkers can be assessed using statistical tests traditionally used to assess clinical investigations such as sensitivity, specificity, positive predictive value, negative predictive value, and area under a receiver operator curve [7]. In addition, if a biomarker is to be used in routine healthcare, a positive net health impact must be demonstrated, including assessment of factors such as cost-effectiveness and impact on quality of life and disease severity [7].

**Fig. 1:**
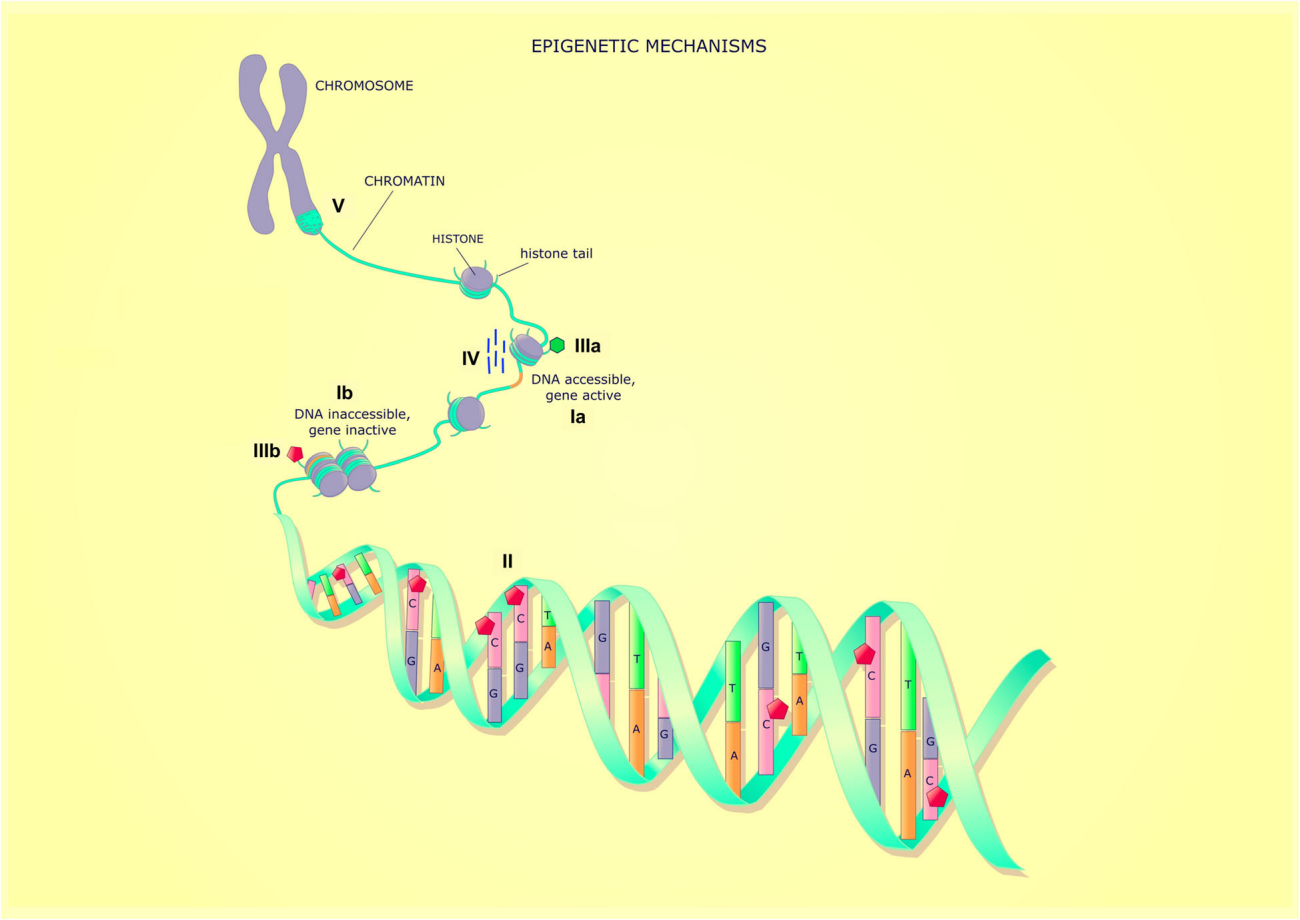
Summary of epigenetic mechanisms. There are multiple epigenetic processes that interact to determine whether DNA (Ia) open and accessible to transcription or (IIb) closed and inaccessible to transcription. Epigenetic processes include (II) DNA methylation, which is the methylation of cytosine residues in CpG dinucleotides, (IIIa) histone tail modifications which can facilitate accessible DNA (histone acetylation and methylation) or (IIIb) histone tail modifications which are associated with inaccessible DNA (histone deacetylation and demethylation), (IV) non-coding RNA which interact with DNA and/or other epigenetic processes, and (V) higher-order chromatin structure (image modified from [9])

Epigenetic processes regulate gene expression, without changes in the underlying DNA sequence [8]. These are tissue-specific (i.e. the epigenetic profile of leucocytes will be different to that of bronchial epithelial cells), generally persist during cell replication (are hence heritable), and maybe influenced by the environment. A recent all-encompassing definition of epigenetics is “modifications of DNA or associated factors that have information content, other than the DNA sequence itself, are maintained during cell division, are influenced by the environment, and cause stable changes in gene expression” [8]. There are several different epigenetic mechanisms (DNA methylation, histone modification, non-coding RNA, and higher-order chromatin structure) which regulate gene expression via modifying the accessibility of DNA to transcription and other regulatory factors (see Fig. 1) [8]. Epigenetic processes do not work independently; rather, they interact to determine gene expression. DNA methylation is the best understood and most useful epigenetic marker to study human disease due to stability over time and ease of measurement [8]. The most abundant form of DNA methylation in the genome involves the addition of a methyl group (CH_3_) to the fifth carbon of a cytosine nucleotide within a cytosine-phosphate-guanine dinucleotide (CpG) [10]. CpG sites are found throughout the genome in low density and are usually highly methylated leading to gene inactivation. They can be found in gene regions such as the promoter (including transcription start sites (TSS), 5′ untranslated region (UTR)), gene body (exons, introns), 3′ UTR, and intergenic regions. A minority of CpG sites are located in highly dense areas called CpG islands, and these are located in the promoter regions of around 70% of all genes [11]. CpG islands usually have a low level of DNA methylation although this does not necessarily mean the gene will be highly expressed as other steps such as transcription factor binding are required for gene expression. Whilst at the level of an individual allele CpG methylation is a binary state (i.e. the CpG is either methylated or not), the DNA methylation level of a sample will range from 0-100%. This is because samples will contain a number of different alleles and will frequently involve multiple different cell types each of which has a unique methylation pattern. Hence, if a sample has a 50% methylation level, this could be because 50% of the alleles in the sample are methylated or because one cell type makes up 50% of the sample, and 100% of the alleles in that cell type are methylated and 0% of the alleles in other cell types are methylated. An individual’s DNA methylation profile is influenced by their underlying genetic makeup and both antenatal and postnatal environmental exposures [12, 13].

As disease outcomes are often thought to be due to a combination of genotype, environmental exposures, and the interaction of the two [14], DNA methylation patterns are potentially ideal biomarkers. Studies attempting to identify DNA methylation biomarkers compare methylation patterns between groups of patients who differ based on the outcome of interest. DNA methylation can be assessed at specific CpG sites of interest or using an epigenome-wide association study (EWAS) approach. The most commonly used product for EWAS is the Illumina Infinium Methylation Array which has evolved from assessing 27,000 (27K array) CPG sites to 450,000 (450k) and now over 850,000 (referred to as the EpicArray). In either design, the average methylation at each CpG site is compared with the aim of identifying statistically significant differential methylation between the two groups, referred to as a differentially methylated position (DMP).

The aim of this review is to summarise the evidence regarding the use of DNA methylation as predictive biomarkers in children with a diagnosed disease. This includes prediction of disease severity, treatment response, or development of complications. Studies examining cancer have not been included as reviews in this area have been recently published [15–17]. In addition, cross-sectional association studies which attempt to link DNA methylation to current disease state have been excluded, as this review focuses on studies which predict future outcomes in children as these offer greater insight as to the predictive ability of DNA methylation profiles in development of disease.

## Evidence Reviewed

Articles were identified via the search strategy in Table 1. A number of studies using DNA methylation as a biomarker in children have been completed (summarised in Supplementary Table 1). Included studies examine whether DNA methylation can predict disease severity [18–26], treatment response [27–34], or development of a complication [35–41].

**Table 1:** Search strategy to identify articles

Database searched	Pubmed
**Dates**	All articles until 5.12.2019
**Search terms**	DNA-methylation* AND (prognosis OR survival OR outcome* OR progression OR deterioration OR pathophysiology OR physiopathology OR morbidit* OR mortalit*) AND (predict* OR response OR risk) AND (newborn* OR baby OR babies OR neonat* OR infan* OR toddler* OR pre-schooler* OR preschooler* OR kindergarten OR boy OR boys OR girl OR girls OR child OR children OR childhood OR adolescen* OR pediatric* OR paediatric* OR youth* OR teen OR teens OR teenage*) AND (NOTNLM OR publisher[sb] OR inprocess[sb] OR pubmednotmedline[sb] OR indatareview[sb] OR pubstatusaheadofprint)

### Neurocognitive impairment in preterm infants

Premature infants are known to have an increased risk of later life neurocognitive impairment. Two studies have attempted to identify DNA methylation biomarkers of future cognitive impairment in premature infants. Tilley et al. [35] examined genome-wide methylation, using the 450K array, in a homogenised sample of chorionic placental tissue of 84 extremely premature infants (born at < 28 weeks gestation). At 10 years of age, participants underwent a cognitive assessment using the School-Age Differential Ability Scales–II. Using logistic regression analysis, they identified 17 probe sites, corresponding to 16 genes involved in neuronal development and function, where increased DNA methylation was associated with cognitive impairment at 10 years. Ten of the probes were located in the gene body, 3 in the promoter region, 3 in the 5′UTR, and 1 in the 3′UTR. The effect size was modest with odds ratios (OR) for these associations between 1.04 and 1.09. For each of the genes, a 1% increase in methylation at their respective probe site resulted in a 4–7% increase in the odds of cognitive impairment. There was evidence of a dose-response relationship, with higher levels of methylation being associated with greater impairment. Arpon et al. [36] examined 22 full-term infants and 24 premature infants (< 34 weeks gestation). They performed an EWAS using a 450K array on whole blood samples collected at 12 months of age. Neurodevelopmental outcome at 2–3 years of age was assessed using the Bayley Scale of Infant Development. Correlations were assessed using Pearson or Spearman tests, and linear regression was performed where significant correlations existed. Correction for multiple testing was undertaken using the Benjamini-Hochberg procedure. They found methylation of a single CpG site located in the 5′UTR of *SLC6A3* showed the greatest difference between preterm and term infants (increased methylation in preterm) and that increased methylation in both term and preterm infants was associated with significantly poorer motor function (*r* = − 0.55, *p* = 0.0001) and mental function (*r* = − 0.44, *p* = 0.0028). The sites of differential methylation identified in each of these studies did not overlap, which may reflect differences in sample size, the biospecimen used for methylation analysis, outcome assessment tool, and age at both methylation analysis and outcome assessment.

### Asthma and allergy

In a large study examining 65 patients with food allergy, Martino et al. [18] assessed DNA methylation in naive CD_4_ T cells at enrolment and then 2–4 years later. Subjects were aged between 11 and 15 months at enrolment and defined as being food allergic based on a positive skin prick test and clinical reactivity. At reassessment, patients were classified as having persistent food allergy (based on oral food challenge) or resolved allergy. The investigators examined whether the change in DNA methylation profiles from baseline to reassessment could predict the likelihood of persistent allergy, and they focused on 26 loci which were demonstrated to be differentially methylated between patients with and without food allergy. They found that in 24 of 26 loci, there was a significant change (adjusted *p* value < 0.05) in methylation from baseline to follow-up in patients with persistent allergy, whereas methylation was stable in patients whose allergy resolved. In the group with persistent allergy, the maximum change in methylation at an individual locus between baseline and follow-up was 17.2%. Fu et al. [42] studied methylation in the 5′UTR of β2 adrenoreceptor (*ADRB2*) in 182 children with asthma aged between 5 and 12 years. Blood was collected at enrolment, and patients were followed up for 12 months. Parent-reported symptoms were used to calculate an asthma severity score, which was used to classify patients into mild or severe disease. Multivariate logistic regression (adjusted for age and gender) was used. DNA methylation was classified as ‘low,’ ‘intermediate,’ and ‘high,’ and asthma as ‘mild’ or ‘severe.’ In a dose-dependent manner, increasing methylation of *ADRB2* was associated with more severe asthma, with those with intermediate methylation having an OR 4.11 (95% CI 1.58–10.73) for severe asthma and those with high methylation having an OR 7.63 (95% CI 3.02–19.26). The median methylation level in the severe asthma group was 1.14% (range 0.2 to 3.6%) and in the mild asthma group was 0.81% (0 to 2.4%). They also examined nitrous oxide exposure and found that in the highly exposed group, those with *ADRB2* hypermethylation had an OR of 4.59 (95% CI 1.03–20.55) of having severe asthma.

Two studies have investigated whether DNA methylation markers can predict response to corticosteroids during an acute asthma exacerbation. Xiao et al. [27] studied Vanin-1 (*VNN1*) methylation in nasal epithelial cells, a surrogate for lower airway epithelium, obtained in 18 children aged 5–18 years at presentation to hospital with an acute asthma exacerbation and again after 12–24 h. *VNN1* was identified as a gene of interest via an initial gene expression investigation of > 20,000 genes. Patients were treated with a standardised asthma protocol which included criteria for discharge. Good corticosteroid response was defined as a length of hospital stay ≤ 24 h, whereas poor response was defined as a length of stay > 24 h. Those who had a good response to corticosteroids had an increase in methylation at a single CpG, located in the promoter region of *VNN1*, whereas those that did not respond to corticosteroids had evidence of decreased methylation, although the raw figures were not reported. When assessed using Fischer’s exact test, the differences were significant (*p* = 0.003). At the same hospital, Zhang et al. [28] used the same design but assessed genome-wide methylation using the 450K array. Their study involved 33 children presenting with acute asthma exacerbations aged 5–18 years. They used linear regression and surrogate variable analysis to correct for confounders, as well as correcting for multiple testing. They found hypermethylation at 1 site in the promoter of lactate dehydrogenase C (*LDHC*) at presentation was associated with poor treatment response. The mean (standard error) methylation level was 87.7% (2.1%) in the poor response group, and 78.6% (2.4%) in the good responders (*p* = 0.007). A major strength of the two studies looking at treatment effect was the use of nasal epithelial cells to assess DNA methylation, which is a better surrogate for bronchial epithelial cells than peripheral blood [43].

### Psychiatric disorders

Another area which has been widely studied in psychiatric disorders. In a group of 164 patients with 22q11 microdeletion syndrome, who are at an increased risk of psychiatric disorders, Starnawska et al. [38] performed an EWAS using the 450K array on neonatal blood spot samples. The later development of a psychiatric disorder was assessed by examining the Danish national psychiatric registry at a mean age of 14.7 years. Linear regression was used to adjust for confounders such as sex and age. A total of 9 differentially methylated probes were significantly associated with development of a psychiatric disorder (unadjusted *p* value < 10^−6^). The effect size of each individual CpG was not described. The probes were located in the promoter region of associated genes (4/9), intergenic regions (3/9), and the gene body (2/9). A further 9 probes associated with intellectual disability, 3 with schizophrenia, 2 with behavioural disorders, and 8 with development disorders. The individual effect size was not detailed for any of these associations. The most common location of probes associated with intellectual disability was the promoter (4/9), and for development disorders, it was the body (4/9). Several studies have examined whether DNA methylation biomarkers can predict response to treatment of psychiatric conditions. Gasso et al. [29] examined whether methylation at 7 CpG sites in the promoter region of 5-hydroxytryptamine receptor 1B (*HTR1B*), measured in blood, could predict treatment response to 12 weeks of the selective serotonin reuptake inhibitor medication, fluoxetine. They studied 83 children aged between 10 and 17 years who were treated with fluoxetine due to major depressive disorder, obsessive-compulsive disorder, or generalised anxiety disorder. Subjects completed multiple validated scales which measure disease severity, and the change in these scales across 12 weeks was used to define treatment response. Pearson’s test was used to assess correlation, and a Bonferroni correction for multiple testing was performed. When the methylation level at the 7 CpG sites was averaged, they found a moderately strong inverse relationship with higher methylation level associated with reduced fluoxetine response (*r* = −0.335; *p* = 0.004). The methylation level for the two groups was not reported. In a study of 116 children aged 6–13 years with an anxiety disorder, the response to cognitive behaviour therapy (CBT) was assessed [31]. Using buccal swab samples, targeted analysis of methylation of 6 CpG sites in the promoter region of the serotonin transporter (*SLC6A4*) was performed pre- and post-treatment with CBT. Treatment response was based on whether the anxiety disorder had resolved. Subjects with a sustained CBT response (measured 6 months post-treatment) were more likely to have increased methylation at a single CpG during treatment (+ 3.48%), whereas non-responders were more likely to decrease (− 5.44%), and this relationship was significant when corrected for multiple testing (*p* = 0.002). In another study also examining buccal swabs from children (*n* = 98) with anxiety disorders, and their response to cognitive behaviour therapy, DNA methylation of CpG sites in the promoter region of FK506 binding protein 5 (*FKBP5*) and glucocorticoid receptor (*GR*) was assessed before and immediately after treatment [30]. The children were aged between 5 and 18 years. Treatment response was defined as a change in primary anxiety disorder severity. No significant effect of *FKBP5* or *GR* methylation was found. In a study of 111 children, mean age 9.25 years, with attention deficit hyperactivity disorder (ADHD) who were treated with methylphenidate, dopamine transporter (*DAT1*), and dopamine receptor D4 (*DRD4*), promoter methylation was assessed in blood samples collected pre-treatment to see if this predicted treatment response [32]. The diagnosis of ADHD was based on fulfilling international criteria, and treatment response was based on change in a validated ADHD severity questionnaire across 6 weeks. Spearman’s rank correlation test was used to assess correlations. They found lower mean *DAT1* methylation was associated with greater treatment response (rho = − 0.222, *p* = 0.019) although the mean methylation levels were not reported.

### Neonatology

Three studies have attempted to identify DNA methylation biomarkers of severity of neonatal abstinence syndrome (NAS). Wachman et al. [20] analysed opioid receptor mu 1 (*OPRM1*) promoter methylation in the saliva of 58 newborns and the relationship with NAS severity. NAS severity was primarily defined based on the need for pharmacological treatment, which was determined based on a standardised protocol. They used linear and logistic regression models as well as correction for multiple testing using the Benjamini and Hochberg method. The study found infants needing pharmacological treatment for NAS had higher methylation at several CpGs: CpG − 18 (11.4% vs 4.4%, *p* = .001), CpG − 14 (46.1% vs 24.0%, *p* = .002), and CpG + 23 (26.3% vs 12.9%, *p* = .008). The same group of authors conducted another study with the same methods and research questions in a group of 86 infants with NAS. They did not replicate the finding with regard to CpGs − 18, +23, but did find that hypermethylation of CpG − 14 was associated with greater severity of NAS measured by need for 2 medications to control symptoms (adjusted difference = 4.9% (95% CI 1.8–8.1%), *p* = 0.0030) [19]. In a study of 21 babies, McLaughlin et al. [21] also used early life saliva swabs and analysed methylation in ATP binding cassette subfamily B member 1 (*ABCB1*) promoter, cytochrome P450 family 2 subfamily D member 6 (*CYP2D6*) exon 1, and *OPRM1* promoter and exon 1. They used a standardised protocol for monitoring NAS severity. Unlike the studies by Wachman et al., they did not find any significant effect. A potential explanation is the smaller sample size of 21, as compared to 58 and 86 in the positive studies.

In a group of 20 premature infants, DNA methylation in the *GR* promoter was analysed in blood from day 4 of life [22]. The outcome of interest was need for treatment with glucocorticosteroids for treatment of lung disease of prematurity or late-onset circulatory collapse, although criteria for exactly when glucocorticosteroids were prescribed were not detailed. Using a logistic regression model, they found a weak association between methylation at a single CpG site and need for treatment with glucocorticosteroids (likelihood ratio 3.889, *p* = 0.0486). Dhas et al. [37] investigated 51 newborns with sepsis which was defined as clinical features and presence of biochemical markers. They analysed global DNA methylation in whole blood and using independent samples *t* tests found those who developed renal failure as a complication of sepsis had slightly higher global methylation levels (2.88% vs. 2.39%, *p* = 0.02).

### Inflammatory bowel disease

Two studies have investigated children with inflammatory bowel disease (IBD). Howell et al. [23] used intestinal biopsy samples from 109 children with IBD and performed an EWAS to identify biomarkers which predicted disease severity over the following 18 months. Disease severity was based on clinical factors such as requirement for treatment with biological agents and time to third treatment escalation following diagnosis. They also evaluated genome-wide gene expression. Differential methylation analysis was performed using limma for DNA methylation data, with a significant false discovery rate [FDR] of < 0.01. They identified a DNA methylation profile that was associated with increased disease severity, as well as demonstrating a gene expression profile that is associated with severe disease with significant overlap in the genes involved. A strength of this study was using samples directly from the tissue of interest. Another study used blood samples collected at diagnosis and the EpicArray to perform an EWAS on 164 children with Chron’s disease [24]. They measured disease severity by considering the time to progress to complicated disease and attempted to identify DMP using linear mixed-effects models and the Bonferroni correction for multiple testing. They were unable to find any CpG sites that were associated with disease severity.

### Idiopathic scoliosis

Meng et al. studied patients with idiopathic scoliosis and the risk of progression of the scoliosis curve beyond 30°, which is a marker of severity. They identified CpG sites of interest in blood, by first investigating two female twin pairs with idiopathic scoliosis who were discordant for curve progression. The top 4 DMP were chosen for replication in a cross-sectional cohort of 92 adolescents who were divided based on the severity of scoliosis at presentation, and only one CpG site (cg01374129) remained a significant DMP. This CpG site is not associated with a gene. They then tested the performance of methylation at this individual CpG in a cohort of 276 adolescent subjects who had mild scoliosis at presentation and then were divided based on whether their scoliosis progressed prior to skeletal maturation. They used the Mann-Whitney *U* test to compare the mean methylation level and progression status. Mean methylation at cg01374129 was lower in the group that progressed (approximately 10% vs. 16%, *p* < 0.0001), and when a receiver operator curve was calculated, the area under the curve was 0.827 (95% CI 0.780 to 0.876), with a sensitivity of 76.4% and a specificity of 85.6% at a cutoff level of 15.1%. Two studies have attempted to identify biomarkers of development of chronic pain complications post-scoliosis surgery. In a study of 128 adolescent patients with idiopathic scoliosis undergoing scoliosis surgery, *OPRM1* promoter methylation was assessed at 22 CpG sites in blood collected during the operation [40]. Acute and chronic pain outcomes were assessed using internationally validated definitions and were dichotomised for analysis. Linear regression models were used for analysis with significance set at *p* < 0.05. There was no correction for multiple testing. After adjustment for preoperative pain score and morphine consumption over postoperative days 1 and 2, higher *OPRM1* promoter methylation at 3 CpG sites was associated with increased acute postoperative pain. The strength of the association was weak for two CpG sites with a 1% increase in DNA methylation associated with regression coefficients of 0.921 and 1.864; however, one CpG had a regression coefficient of 17.736. When chronic postsurgical pain was assessed, there was significant different methylation at two CpG sites. The OR for these associations were relatively weak: 1.069 (95% confidence interval 1.022–1.119) and 1.037 (1.000–1.075). Once again, adjustment was performed for preoperative pain and postoperative morphine consumption. The two CpG sites did not overlap with those associated with acute postoperative pain. The authors provided further evidence for the role of *OPRM1* methylation via using functional genomic analysis from publicly available databases which showed increased methylation would lead to reduced gene expression and reduced response to opioids. In a subsequent study, the same authors assessed 46 adolescents undergoing scoliosis surgery. They performed an EWAS using the EpicArray on peripheral blood samples. For this study, they used medication use and questionnaires to assess pain severity. Surrogate variance analysis was used to control for confounders, and adjusted linear regression models were used to test for DMP. They identified 637 significantly differentially methylated probes associated with chronic postsurgical pain (*p* < 0.05). The DMPs were most commonly found in the promoter region (23.31%) and gene bodies (36.34%). The 637 DMPs were associated with 310 genes and which are associated with pathways such as GABA receptor signalling, protein kinase C signalling, dopamine receptor, and cyclic adenosine monophosphate-mediated signalling.

### Other conditions

A number of other conditions have been investigated by single studies. A single study has investigated 68 children with juvenile idiopathic arthritis [26]. Patients were stratified based on relapsing or sustained remission following cessation of anti-tumour necrosis alpha treatment. The 450K array was performed on CD_4_ T cells collected as anti-tumour necrosis alpha treatment was stopped. Weighted gene coexpression network analysis was used to identify differential methylation. This analysis examines correlated CpGs rather than individual CpGs and reports differentially methylated regions (DMR). They identified 5 DMRs between the two groups. The DMRs were associated with genes implicated in T cell activation, and this relationship was confirmed in subsequent gene expression and protein analysis experiments, with elevated markers of T cell activation in those who did not have sustained remission post-therapy cessation. Kuo et al. [33] studied 36 patients with Kawasaki disease and assessed the relationship between Fc fragment of IgG receptor IIa (*FCGR2A*) promoter methylation and response to treatment with intravenous immunoglobulin (IVIG), based on defervescence within 48 h. The sample source for methylation analysis was not reported. Mean methylation levels between responders and non-responders were compared using the chi-square test. They identified 5 CpG sites where increased methylation was associated with non-response to IVIG. At the 5 CpG sites, the difference in mean methylation level between the two groups ranged from 9.75 to 18.94%, and at all sites, the relationship was strong (*p* value < 0.0001). Yang et al. [34] studied 20 patients with infantile spasms and whether global DNA methylation in blood collected prior to treatment starting was associated with response to treatment with ACTH (based on change in seizure frequency). Mean methylation between the responders and non-responders was compared using the unpaired Student’s *t* test. No relationship between mean DNA global methylation level and ACTH treatment effect was identified. In a group of 97 patients with Beckwith-Wiedemann syndrome, Gaston et al. [41] examined the relationship between *KCNQ1OT* and *H19* methylation and risk of tumour development (a known complication of Beckwith-Wiedemann syndrome). They used blood samples, and in some patients, tissue samples from affected organs were also obtained. Using the Kaplan-Meier method, abnormal *H19* methylation (defined as a methylation index > 60%) was strongly associated with an increased risk of tumour development (34.6% vs. 4.2%, hazard ratio 10, *p* < 0.0001).

## Discussion

Several studies have investigated the use of DNA methylation as a biomarker to predict future health outcomes in children. In support of the notion that DNA methylation is a viable biomarker, 22 of the 25 studies report a positive finding, although that may represent publication bias [44]. Unfortunately, due to issues with chosen outcomes, tissue-specific samples, accounting for sample cell type heterogeneity, lack of appropriate statistical testing, small effect sizes, limited validation, and no assessment of net health impact, the goal of DNA methylation-based biomarkers being used in clinical care for non-oncological paediatric diseases remains distant.

One of the most important aspects of any biomarker study is to ensure that the outcome of the study is robust and clinically relevant. A number of studies have used validated and widely accepted tools for assessing the outcome, such as the use of the Bayleys Scale of Infant Development for assessing neurocognitive development. Where possible, gold standard outcome measures should be used as they are more robust than other outcome measures such as parent-reported asthma symptoms, which are open to error [45]. This is of particular relevance for studies examining severity of disease where the study uses doctor prescription of a medication as a marker of severity (i.e. corticosteroids in prematurity or biological agents in IBD). Such endpoints are valid, but only if the indications for such treatments are clearly explained, in keeping with international prescribing practices and are true surrogates for disease outcomes. If not, any change seen may reflect the alternative practice of those involved in the study and may not be generalizable to a wider population.

Another important consideration is the tissue source for DNA methylation analysis. As a large proportion of methylation variation is tissue-specific [8], it is desirable (where possible) to analyse samples from the target tissue [46], for example, using intestinal biopsies to identify biomarkers in IBD [23] rather than using blood samples which were used in the majority of studies. It can be difficult to obtain samples from the tissue of interest in living humans, and particularly children as they are less tolerant of invasive testing. Potential solutions involve using non-invasive sampling methods such as induced sputum [47] or urine samples [48] which have successfully been used for DNA methylation analysis [49, 50]. Another potential method for analysing tissue-specific DNA methylation in a relatively non-invasive manner is by analysing cell-free DNA, which is made up of small fragments of DNA that circulate in the blood and are thought to originate from apoptotic/dying cells [51]. The original cell type of cell-free DNA can often be determined through the identification of DNA methylation signatures limited to a specific tissue type [52], and in adult diseases, these may act a biomarker to identify specific health conditions [53]. To date, this approach has largely been applied to early identification of primary (or relapsed) malignancy or antenatal screening for fetal genetic abnormalities [54]. As yet, this approach has not been assessed in children for less pathogenic conditions, but represents a method to potentially sample the tissue of interest from a peripheral blood sample.

Where samples used for DNA methylation analysis involve multiple cell types (i.e. blood, buccal swabs), it is important that cell composition of the sample is accounted for in the analysis [55]. As each cell type will have a unique DNA methylation profile, any differences seen between cases and controls may equally reflect differences in cell composition as bona fide methylation changes associated with disease. A relative weakness of most studies included in this review is that the majority of them (15 of 25) do not account for issues arising from differential cell composition. Six of 25 [24, 29, 35, 36, 38, 39] did account for cell composition, whilst several [18, 23, 26–28] avoided this issue by using a sample that only included one cell type. A differing view is that alterations in cell composition are often an important feature of a specific disease/condition (i.e. inflammation will alter the cell composition of blood) and therefore should not be ‘adjusted for’ in any methylation analysis. Rather, they may provide key insights into the cellular disruption associated with disease pathogenesis.

The statistical analyses used to date limit the clinical applicability of the evidence that has been generated. For a test to be used in clinical practice, the performance should be assessed via statistical measures such as sensitivity, specificity, positive predictive value, negative predictive value, and area under a receiver operator curve. Only one [25] of the included studies used these statistical measures, whilst the majority of studies used statistical tests that describe associations. This may be because the authors are focusing on identifying associations which may inform disease mechanisms and therapeutic targets, but it would be ideal if statistical tests of predictive ability were also used as identification of clinically relevant biomarkers would be extremely useful. Another important statistical consideration is study power. The majority of EWAS studies conducted have had a small sample size, with no power calculation performed, and are likely underpowered [46]. However, recently, a publicly available tool for EWAS power calculation has been developed making it easier to design adequately powered studies, although it is only suitable for use with certain commonly collected specimens [56]. Given current genome-wide methylation assays measure methylation at 850,000 sites, another important statistical consideration is correction for multiple testing and there are many well-established methods for this.

A major limitation to the evidence generated in this field so far is the generally reported small disease-associated effect sizes of differential methylation between groups. These likely arise from a combination of the limited sensitivity and specificity of current methods used to assess DNA methylation, relative to the gold standard whole-genome bisulphite sequencing [57] in combination with the relatively large disease-independent inter-individual variation in methylation at many genomic sites. Weak evidence of associations is unlikely to perform well when aligned against the criteria of a clinically relevant biomarker. In addition, these findings are less likely to be replicated in external cohorts. A lack of replication is another limitation of the current evidence. As mentioned, 22 papers had a significant finding; however, for the majority of these findings, there has been no attempt at replication. One area where there has been an attempt at replication is the role of *OPRM1* methylation in NAS, with two studies finding a significant association [20] and one study finding no association [21]. A number of EWAS have yielded positive results; however, these have not been replicated in either subsequent EWAS or targeted studies.

Despite the limitations of the evidence so far, there is still potential for useful DNA methylation biomarkers to be identified. Whilst most of the issues raised can be overcome by careful study design, the small observed effect sizes may be more difficult. One approach would be to incorporate DNA methylation data into combined predictive models. These models could pool data from multiple ‘omic’ inputs such as the genome (genetic mutations), methylome (DNA methylation), transcriptome (gene expression), and proteome (protein levels). An example of a program which is using the proposed multi-omic approach to better understand disease pathology and predict disease outcomes is the Integrative Human Microbiome Project. The second phase of this project is collecting biospecimens from patients to investigate the onset of inflammatory bowel disease and type 2 diabetes and is analysing host genome, methylome, transcriptome, and proteome as well as other factors such as the microbiome [58]. The omics data could also be integrated with existing diagnostics tests, clinical information, and also information regarding environmental exposures. In a study which examined prognostic biomarkers in multiple cancer types, omic-based biomarkers (including DNA methylation) outperformed existing clinical scores, and a combination of omic and clinical data had the best performance [59]. In another study examining adult patients, the combination of clinical information (via regular clinical assessment), environmental data (obtained using wearable sensors), and proteomics (via assessment of high sensitive C reactive protein) allowed earlier detection of inflammatory diseases such as Lyme disease and also earlier detection of insulin resistance [60]. The integration of the multiple data sources, however, is not without challenges [61]. In addition to demonstrating that such a biomarker performed well from a statistical point of view, a positive net health impact would also need to be demonstrated [7]. However, if these hurdles are overcome, biomarkers encompassing multiple data sources, including DNA methylation, will represent a great advance in health care.

## Conclusions

If the goals of precision health are to be recognised, biomarkers which predict future health outcomes will need to be identified. With the expansion in ‘omics’ technology, there are multiple tools which may aid discovery of such biomarkers. One such tool is epigenetics, in particular DNA methylation, and multiple studies have already undertaken research in this area. Whilst several positive findings have been made, there is a long way to go before these findings can be incorporated into clinical care. To ensure the validity of any future discoveries, researchers should ensure consideration is given to disease outcomes, tissue-specific specimens, adjustment for cell-type heterogeneity, appropriate statistical tests, replication of positive findings, and incorporating DNA methylation data into combined biomarkers. If these factors are considered, DNA methylation biomarkers have the potential to improve the healthcare delivered to children.

## Supplementary information

**Additional file 1: Table 1.** Included articles.

## Data Availability

Not applicable (review article)

## Abbreviations

DNA: Deoxyribonucleic acid
RNA: Ribonucleic acid
CpG: Cytosine-phosphate-guanine dinucleotide
UTR: Untranslated region
TSS: Transcriptional start site
EWAS: Epigenome-wide association study
DMP: Differentially methylated positions
OR: Odds ratio
*ADRB2*: β2 adrenoreceptor
*VNN1*: Vanin-1
*LDHC*: Lactate dehydrogenase C
*HTR1B*: 5-Hydroxytryptamine receptor 1B
CBT: Cognitive behavioural therapy
*SLC6A4*: Serotonin transporter
*FKBP5*: FK506 binding protein 5
*GR*: Glucocorticoid receptor
ADHD: Attention deficit hyperactivity disorder
*DAT1*: Dopamine transporter
*DRD4*: Dopamine receptor D4
NAS: Neonatal abstinence syndrome
*OPRM1*: Opioid receptor mu 1
*ABCB1*: ATP binding cassette subfamily B member 1
*CYP2D6*: Cytochrome P450 family 2 subfamily D member 6
IBD: Inflammatory bowel disease
FDR: False discovery rate
DMR: Differentially methylated regions
*FCGR2A*: Fc fragment of IgG receptor IIa
IVIG: Intravenous immunoglobulin
